# *Acinetobacter* infections prevalence and frequency of the antibiotics resistance: comparative study of intensive care units versus other hospital units

**DOI:** 10.11604/pamj.2016.23.191.7915

**Published:** 2016-04-15

**Authors:** Jean Uwingabiye, Mohammed Frikh, Abdelhay Lemnouer, Fatna Bssaibis, Bouchra Belefquih, Adil Maleb, Souhail Dahraoui, Lahcen Belyamani, Abdelouahed Bait, Charki Haimeur, Lhoussain Louzi, Azeddine Ibrahimi, Mostafa Elouennass

**Affiliations:** 1 Service de Bactériologie, Hôpital Militaire d'Instruction Mohammed V, Rabat, Maroc; 2 Equipe de recherche: Épidémiologie et Résistance Bactérienne (ERB), Université Mohammed V, Faculté de Médecine et de Pharmacie, Rabat, Maroc; 3 Service des Urgences, Hôpital Militaire d'Instruction Mohammed V, Rabat, Maroc; 4 Services des Réanimations, Hôpital Militaire d'Instruction Mohammed V, Rabat, Maroc; 5 Laboratoire de Biotechnologie, Université Mohammed V, Faculté de Médecine et de Pharmacie, Rabat, Maroc

**Keywords:** Acinetobacter, prevalence, antibiotic resistance intensive care units

## Abstract

**Introduction:**

This study aims to determine the *Acinetobacter* sp clinical isolates frequency and its antibiotic susceptibility pattern by comparing results obtained from the Intensive Care Units (ICUs) to that of other units at the Mohammed V Military Teaching Hospital in Rabat.

**Methods:**

This is a retrospective study over a 2-years period where we collected all clinical isolates of *Acinetobacter sp* obtained from samples for infection diagnosis performed on hospitalized patients between 2012 to 2014.

**Results:**

During the study period, 441 clinical and non-repetitive isolates of *Acinetobacter* sp were collected representing 6.94% of all bacterial clinical isolates (n = 6352) and 9.6% of Gram negative rods (n = 4569). More than a half of the isolates were from the ICUs and were obtained from 293 infected patients of which 65, 2% (191 cases) were males (sex ratio = 1.9) and the median age was 56 years (interquartile range: 42-68 years). *Acinetobacter* clinical isolates were obtained from respiratory samples (44.67%) followed by blood cultures (14.51%). The resistance to ciprofloxacin, ceftazidime, piperacillin / tazobactam, imipenem, amikacin, tobramycin, netilmicin, rifampicin and colistin was respectively 87%, 86%, 79%, 76%; 52%, 43%, 33% 32% and 1.7%. The difference in resistance between the ICUs and the other units was statistically significant (p <0.05) except for colistin, tetracycline and rifampicin.

**Conclusion:**

This paper shows that solving the problem of prevalence and high rate of multidrug resistant *Acinetobacter* infection which represents a therapeutic impasse, requires the control of the hospital environment and optimizing hands hygiene and antibiotics use in the hospital.

## Introduction

*Acinetobacter* is a non-fermenting Gram negative coccobacillus with a high capacity to colonize the human body and the environmental reservoirs [1]. It has become over the past three decades a major associated care infections agent with a high morbidity and a high mortality rate especially in immunocompromised patients ranging from 26.5 to 91% [2–5]. In Morocco, a retrospective study [6] from 2002 to 2005 showed that this bacterium represented 13.63% of clinical isolates from blood cultures in the intensive care units (ICUs) [6] and in another Moroccan study [7], it represented 6.74% of all Gram-negative bacilli. The *Acinetobacter* infection prevalence is variable depending on the geographical localization and the patient's socio-economic status [8–10]. In an international study in ICUs, the *Acinetobacter* infections rate was 19.2% in Asia; 17.1% in Eastern Europe; 14.8% in Africa; 13.8% in Central and South America; 5.6% in Western Europe; 4.4% in Oceania and 3.7% in North America [10]. It is 15% in South African HIV-positive patients [8] and 13% in Canadian burn care units [9]. In our region, no studies on *Acinetobacter* prevalence have been performed. *Acinetobacter* is an opportunistic pathogen known for its intrinsic resistance to antibiotics and greater ability to rapidly acquire resistance genes as mobile genetic elements (plasmids, transposons, integrons cassettes and insertion sequences) [11–13]. Multidrug resistant (MDR) *Acinetobacter baumannii* is becoming a global threat with a therapeutic impasse increasingly described in literature [14–16]. Indeed this organism generally has resistance to several antibiotics. According to the literature data, the resistance rate varies from 31.8 to 92.1% to ceftazidime; 8.8 to 89.9% vs imipenem, from 12.2 to 89.9% vs Piperacillin / Tazobactam, from 28.8 to 91.6% vs fluoroquinolones and 30 to 90.3% vs aminoglycosides [8, 17–20] but colistin is often the only effective treatment option whereas some *Acinetobacter* strains develop resistance to colistin [8, 18–21]. Resistance to colistin was estimated to 5.3% in the United States [21]; 2.7% in South Africa [8]; 1.2% in India [20] and 0.9% in Tunisia [19] and 0.5% in Saudi Arabia [18]. In Morocco, the *Acinetobacter's* antibiotic resistance rates were of 50.3 to 68.7% for ceftazidime, 23.8 to 42.6% for the imipenem, 17 to 77.5% for aminoglycosides, 65 to 68% for ciprofloxacin and no clinical isolates were resistant to colistin [6, 7, 22] however these data were inadequate and old. The purpose of this study was to accurately determine the prevalence rate of infections and antibiotic resistance level in clinical isolates of *Acinetobacter* by comparing data from the intensive care units versus other units of the Mohammed V Military Teaching Hospital (HIMMV).

## Methods

### Setting

This study was conducted in HMIMV, a teaching hospital with 700-bed located in Rabat, Kingdom of Morocco. The hospital has different departments mainly 2 intensive care units (medical and surgical) with 10 beds each, a center for burns treatment, surgical and medical units, as well as laboratory and imagery departments.

### Type of studies

This retrospective study was conducted by the laboratory of medical microbiology over a 2-years period. Clinical isolates were collected from diagnosis samples performed on patients who were hospitalized in different units of the HMIMV from April, 1^st^, 2012 to April, 1st^st^, 2014.

### Isolation cultures and antibiotic susceptibility

Isolation of *Acinetobacter* was performed on blood agar and bromo-cresol purple lactose agar and identification of clinical isolates was performed by classical bacteriological techniques (direct examination, biochemical test of orientation) and biochemical characters using API20NE galleries (Biomérieux, Marcy l'Etoile, France). The study of antibiotic susceptibility was performed by the disc diffusion method on Mueller-Hinton agar plates, and interpreted as recommended by the antibiogram committee of the French Society of Microbiology in their 2014 recommendations. The antibiotic discs tested were: ticarcillin, ticarcillin-clavulanate, piperacillin, piperacillin-tazobactam, cefepime, ceftazidime, imipenem, amikacin, gentamicin, tobramycin, netilmicin, ciprofloxacin, sulfamethoxazole -trimethoprime and colistin. The reading of the antibiograms was performed using the OSIRIS expert system. The resistance to colistin was confirmed by the determination of the Minimum Inhibitory Concentrations (MICs) using the E-test method (Biomérieux, Marcy l'Etoile, France) according to the manufacturer′s recommendations. All isolates of *Acinetobacter* resistant to three or more classes of antibiotics represented by piperacillin / tazobactam, ceftazidime, imipenem, ciprofloxacin, aminoglycosides and colistin were considered as MDR [18, 21, 23].

### Statistical analysis

The statistical analysis was performed using the SPSS Statistics 17.0. The Chi 2 test was used to compare the percentages of *Acinetobacter* infection prevalence, resistance rates and MDR between ICUs and the other units. The p values less than 0.05 were considered statistically significant.

## Results

### Characteristics of *Acinetobacter*′s clinical isolates

During the study period, 441clinical isolates of *Acinetobacter* were collected, representing 6.94% of all bacterial isolates (n = 6352) and 9.6% of all Gram-negative bacilli (n = 4569) throughout the hospital. These isolates were obtained from 293 *Acinetobacter* infected patients of which 65.2% (191cases) were males, so a sex ratio M/F of 1.9. These patients represented 8, 2% of all patients having a bacterial infection (n = 3565). The median age of *Acinetobacter* infected patients was 56 years (interquartile range: 42-68 years) and the distribution by age showed that 64.9% of the isolates came from patients aged between 18-64 years; 31.3% aged 65-101 years and 3.8% of patients ≤ 17 years. Site sampling analysis of the *Acinetobacter's* isolates showed that the proportion of broncho-pulmonary samples was 44.67%, followed by the blood cultures (14.51%), the deep pus (12.47%), the urine (12%), the superficial pus (9%), the catheters (3.85%), the tissue (1.81%) and the puncture liquid (1.59%) (Table 1). Isolates of *Acinetobacter* under study, 358 (87.1%) were *Acinetobacter baumannii*, and 4 (1%) were *Acinetobacter lwoffii*. The breakdown by department analysis showed that 54.9% of clinical isolates were obtained from the ICUs, 36.7% and 8.4% from the medical and surgical units respectively. In the ICUs, the isolates of collected *Acinetobacter* strains (n = 242) represented 24.85% of all isolates (n = 974) and 31.5% of all Gram-negative bacilli (n = 769). They were collected from 156 patients infected with *Acinetobacter*. For these patients 61.53% were male with a sex ratio of 1.6 and represented a 49.2% of patients with bacterial infection (n = 317). In the other units, 199 isolates of *Acinetobacter* were collected, representing 3.7% of all isolates (n = 5378) and 5.23% of Gram-negative rods (n = 3800) obtained in these units. These isolates were obtained from 137 infected patients. Among these patients, 69.3% were male, so a sex ratio equal to 2.3 and represented a rate of 4.2% of whole patients with bacterial infection (n = 3248). The difference in the prevalence of *Acinetobacter* related-infections between the ICUs and the other units was statistically significant (p <0.001).

**Table 1:** Repartition of clinical isolates of *Acinetobacter* by units and sampling site

Samples nature	ICUs (%)	Other units (%)	Total (%)
Bronchopulmonary	64,8	14	44,67
Blood cultures	15,7	12,7	14,51
Deep pus	5	23,6	12,47
Urine + Urinary catheters	6,6	22,3	12,02
Superficial pus	3,3	19,7	9,07
Vascular catheters	2,1	4,5	3,85
Tissues and biopsies	1,7	1,3	1,81
Fluids of puncture	0,8	1,9	1,6

### Antibiotic susceptibility

**Figure 1:**
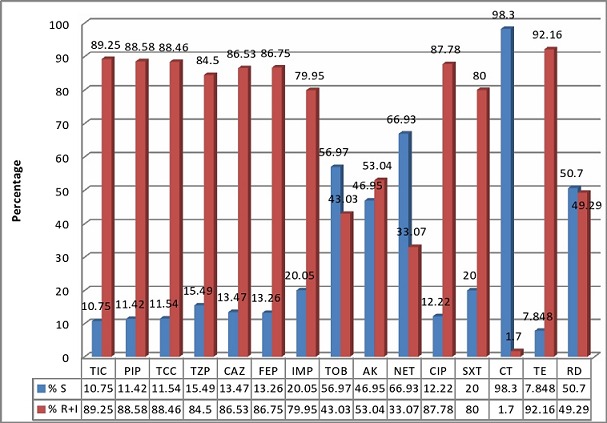
Susceptibility pattern of clinical isolates of *Acinetobacter*

Figure 1 shows the overall sensitivity patterns of clinical isolates of *Acinetobacter* sp with the resistance rate to colistin of 1.7%. The resistance rates difference between the ICUs and the other units was statistically significant (p< 0.05) except for colistin, tetracycline and rifampicin (Table 2). The resistance′s phenotypes to beta-lactams have been dominated by the carbapenemase or alteration of porins (63.1%). The MDR′s percentage was 77.5% for the all *Acinetobacter* clinical isolates according to the used criteria. The MDR distribution rates in accordance with each kind of sample are shown in Table 3. The MDR′s rate was 92.6% in ICUs and 75.3% in other units. The MDR's rate difference between the ICUs and the other units was statistically significant (p <0.001).

**Table 2:** Comparison of *Acinetobacter* resistance rates in ICUs versus other units

Antibiotics	Resistance rates (in %)			*p*
	Total (n = 441)	ICUs (n = 242)	other units (n = 199)	
Ticarcillin	89,00	98,3	75,46	p< 0.001
Piperacillin	87,56	97,85	72,84	p< 0.001
Ticarcilline/ clavulanic acide	87,41	95,7	76,23	p< 0.001
Piperacillin/Tazobactam	79,15	90,7	61,15	p< 0.001
Ceftazidime	86,03	95,8	72,12	p< 0.001
Cefepime	86,17	94,66	73,76	p< 0.001
Imipenem	76,19	87,7	59,51	p< 0.001
Tobramycin	43,03	44	30,91	p< 0.001
Amikacin	52,28	59,3	42,33	p = 0,027
Netilmicin	33,07	38,4	25,16	p = 0,033
Ciprofloxacin	87,78	96,6	75,00	p< 0.001
Sulfaméthoxazole/Trimetoprim	78,96	87,3	66,88	p< 0.001
Colistin	1,7	1,68	1,79	p = 0,869
Tetracycline	90,89	93,53	87,12	p = 0,146
Rifampicin	32,11	34,3	28,97	p = 0,403
MDR rate	77,5	92,6	75,3	p< 0.001

**Table 3:** Proportion of the MDR *Acinetobacter* clinical isolates by sampling site

Sampling sites	Number of isolates	% MDR
bronchopulmonary	197	89,43
blood culture	64	70,3
deep pus	55	70,9
superficial pus	40	67,5
catheters	17	94,1
urine	53	52,83
Fluids of puncture	7	71,42
tissue	8	75
total	441	77,5

## Discussion

The present study shows that the infection *Acinetobacter 's* prevalence in HMIMV is high with higher rates in ICUs compared to other units (p <0.001). The isolation rate of *Acinetobacter* in the various samples was 6.94%. These results are higher compared to those from the study conducted by Mushtaq and al. (2013) [14] in Pakistan where the isolation rate of *Acinetobacter* species was 4.2% [11]. In an international study on the prevalence of infections in ICUs in 75 countries [10], the isolation rate of *Acinetobacter* (8.8%) was significantly lower than that of the ICUs (24.85%) in our hospital. These clinical isolates represented 9.6% of all gram-negative bacilli in the hospital and 31.5% in the ICUs. On the other hand, a study carried out in an Indian hospital reported a comparable rate as *A. baumannii* constituted 9.4% of all Gram-negative rods throughout the hospital and 22.6% in the ICUs [20]. Our study shows that the frequency and resistance of *Acinetobacter* isolates are increasing in our hospital. Indeed, in 2001, 147 clinical isolates of *A. baumannii* were isolated from all patients hospitalized in our institution and checked for bacterial infection [22] against 441 clinical isolates in our study period; April 1^st^, 2012 to April 1^st^, 2014. This high prevalence observed in our study is probably related to non-compliance with the recommendations for mastery the hospital environment [24], lack in hands hygiene and misuse of antibiotics [25]. Some studies have reported that this microorganism which has emerged worldwide as a pathogen causing serious infections in hospitalized patients has the ability to persist in the environment for a long period of time, colonize patients or healthy subjects and can develop into a true infection at any time [26]. Since hand transmission is a major factor in the spread of this pathogen [24], hand hygiene and disinfection of equipment/environment are the two most important factors to control and prevent the outbreak of an epidemic *Acinetobacter* [24].

In our study, 65.2% of affected patients were male. The predominance of male patients infected with *Acinetobacter* has been verified in other studies but the reason is not justified [2, 3, 5, 11]. The average age of patients in our study was 54 years (2-101 years) with predominance of patients over 17 years; these results are similar to those of many authors [2, 3, 5, 11]. The old age of patients was recognized as an independent risk factor of the acquisition of *A. baumannii* infection [5]. Many authors have reported the predominance of *Acinetobacter* strains in broncho-pulmonary samples [7, 20, 27]. In this study, the main isolation site of these clinical isolates was also broncho-pulmonary (44.67%) followed by blood cultures (14.51%). The *Acinetobacter* spp infections are generally involved in anatomical sites with a high fluid content manifested by pneumonia, bacteremia, urinary tract infection, meningitis and wound infection [1]. Several studies have shown that the high frequency of *A. baumannii* pneumonia is associated with mechanical ventilation [3, 28] resulting in extended stays in ICUs, the rapid development of resistance to commonly used antibiotics and a high mortality ranging from 45.6 to 84.3% according to the authors [3, 28]. The majority of clinical isolates in this study were essentially of *A. baumannii* (87.1%). The research by Chuang and al. (2011) demonstrated that among *Acinetobacter* species, *A. baumannii* is the main cause of *Acinetobacter* infections with the antibiotic resistance rate being very high causing more serious infections than other species of *Acinetobacter* [29]. In general, the *Acinetobacter* isolates are known for their resistance to various antibiotics despite their weak virulence limiting the control and infections treatment due to these microorganisms [1–5]. Our study shows that the rate of antibiotic resistance in our hospital is generally high and variable. Several authors have confirmed the high prevalence of these infections associated with high resistance in ICUs [7, 17, 20, 30]. The high proportion and the high resistance of these microorganisms in ICUs are related to the existence of numerous risk factors associated with *Acinetobacter* infection such as immunocompromised persons, longer duration of stay in hospitals, invasive devices use on patients, the broad spectrum antibiotics therapy, possible and frequent contaminations and cross transmission of this bacteria through environmental reservoirs and hands of healthcare workers [3, 24]. For the beta-lactam antibiotics which are a large family playing an important role in antimicrobial treatment [31], the high resistance of *Acinetobacter* clinical isolates to this class of antibiotics (ceftazidime, cefepime, imipenem and piperacillin / tazobactam) has been described in the literature [31]. In our study, the resistance rate against tested beta-lactam antibiotics ranged from 76% to 89% throughout the hospital, from 87 to 98% in the ICUs and 59-75% in the other units. This rate is comparable to that observed in Asia, where it ranged from 56 to 94% [14, 20, 27, 32]. In a Libyan study, the resistance was of 62.3 to 98.8% throughout the studied hospital, 71.6-100% in the ICUs and from 42.6 to 96.2% in the other units [31]. The resistance to ceftazidime and cefepime was 86%. In the ICUs, the resistance rate to ceftazidime was 95.8% and 72.2% in the other units. This rate is higher than that obtained in the United States and South Africa in a similar study where the resistance to ceftazidime was 52.1% and 68.4% respectively [8, 21]. It ranges from 60 to 92.1% in studies in Asia [14, 20, 27, 32].

Carbapenems (imipenem, meropenem) remain one of the most important therapeutic options for these infections despite the fact that carbapenem-resistant strains are increasing [25]. In our study, the resistance to imipenem was 76.19% throughout the hospital and 87.7% in the ICUs. This rate is lower than that noted in India, where the resistance to imipenem reached 89.6% [20] and higher than those of previous studies in Morocco: 23.6% [22] in 2001, 42.6% in 2005 [7] and those obtained in the United States and Saudi Arabia, where the resistance to imipenem was 44.7% and 61.3% respectively [18, 21]. Carbapenem resistance in *A. baumannii* is often due to the expression of OXA carbapenemase types, Metallo-beta-lactamases (MBL) carbapenemase and the impermeability associated with mutations altering the expression of porins and efflux pumps [3, 30]. In this study, most of the *Acinetobacter* strains showed the phenotype having resistance to beta-lactam antibiotics associated with the expression of carbapenemase or alteration of porins (63, 1%). These results are not in accordance with those found in another study conducted in the same hospital where the penicillinase phenotype and high level cephalosporinase production were predominant with a rate of 33% [22]. In a Tunisian study, the majority of *A. baumannii* trains had the penicillinase phenotype in 26.3% of cases [19]. This emphasizes the alarming increase in resistance to imipenem and the expression of carbapenemase often related to the misuse of this antibiotic class in the clinical departments of our hospital. For the aminoglycosides, netilmicin was the most effective with a resistance rate of 33.07% against 43.03% for tobramycin and 52.28% for amikacin. Our data are different and lower than those obtained by Jaggi and al. (2012) in India; where resistance to tobramycin, gentamicin, netilmicin and amikacin were 80.0, 85.8, 90.3 and 90.3% respectively [19]. The aminoglycosides resistance in *Acinetobacter* spp. involves the production of aminoglycosides modifying enzymes and genes encoding these enzymes can be acquired through plasmids, transposons or integrons [3, 20]. The rate of resistance to ciprofloxacin observed in our study (87.7%). This rate is comparable to that reported in the literature which varies from 28.8 to 91.6% [8, 17–20]. The prescription of this drug in the treatment of *Acinetobacter* infections is rare because of the high resistance to this antibiotic in our institution. Rifampicin was very effective (but less than colistin) with a resistance rate of 32.11%, but the use of this drug in the treatment of *Acinetobacter* infections is limited because Morocco is a country of endemic tuberculosis. Colistin was the most active antibiotic against *Acinetobacter*. In this study, the resistance to colistin was 1.7%.

Some studies have reported that no clinical isolate of *Acinetobacter* was resistant to colistin [13, 22] but the resistance to colistin has been described in India, South Africa and Korea [20, 33]. Several authors confirm that colistin remains the only option for empirical treatment of serious *Acinetobacter* infections in cases where this bacterium is strongly suspected to be resistant to other antibiotics [12, 19]. The mechanism of resistance to this antibiotic is rare and may be explained by the loss of lipopolysaccharide and/or deployment of a system of two-component regulatory PmrAB [34, 35]. Synergy between colistin and rifampicin or anti-Pseudomonas carbapenem is described in some studies [25]. There are no specific recommendations regarding the combination of antibiotics for the treatment of these serious infections due to the lack of prospective comparative clinical trials with a control group [25]. The combination therapy used in ICUs departments of our hospital includes colistin associated with imipenem or rifampicin. The resistance to ceftazidime and imipenem was 86.03% and 76.19% respectively in this study versus 63.3% and 23.3% respectively in 2001. The resistance to colistin increased slightly in our study by 1.7% against 0% in 2001 [22]. This increase in resistance to these antibiotics can be explained by the uncontrolled growth of their consumption in our hospital. However there was a decrease in resistance to tobramycin and sulfamethoxazole / trimethoprim: 70.8% and 83.1% respectively in 2001 [22] as against 43.03% and 78.96% respectively in our study, probably linked to general down use of these categories of antibiotics in our hospital.

The current study demonstrated that the percentage of MDR *Acinetobacter* was 77.4% throughout the hospital and extents 92.6% in ICUs and 75.3% in the other units with a rate difference that was statistically significant between the ICUs and the other units (p <0.001). Depending on the nature of each sampling site, the highest MDR *Acinetobacter* percentage (94.1%) was observed in vascular catheters followed by broncho-pulmonary samples (89.4%) and the lowest MDR *Acinetobacter* percentage (52.83%) was found in urine samples. These results are comparable to conclusions of a Lebanese study where rates of MDR *Acinetobacter* varied between 73.4% and 77.7% [16] but higher than and non-similar to the findings observed in the United States (54%) with higher frequency in both of patients over 65 years old and respiratory specimens [21].

## Conclusion

In this study, we showed that, in our hospital, the frequency and rates of MDR *Acinetobacter* infection are high and could pose a real problem and a management impasse. A strict control of the hospital environment, hand hygiene and optimizing the use of antibiotics is recommended in order to reduce the MDR frequency.

### What is known about this topic

- *Acinetobacter* is a non-fermenting Gram negative coccobacillus with a high capacity to colonize the human body and the environmental reservoirs and it is associated with a high morbidity and a high mortality rate especially in immunocompromised patients.
- *Acinetobacter* is an opportunistic pathogen known for its intrinsic resistance to antibiotics and greater ability to rapidly acquire resistance genes.
- It generally has resistance to several antibiotics and MDR *Acinetobacter baumannii* is becoming a global threat with a therapeutic impasse increasingly described in literature.

### What this study adds

- This is the first study which shows the prevalence rate of infections and antibiotic resistance level in clinical isolates of *Acinetobacter* by comparing data from the intensive care units versus other units in our hospital and in our region.
- This study shows that the frequency and resistance of *Acinetobacter* isolates are increasing in our hospital.
- This is the first Moroccan study that reports the rate of MDR *Acinetobacter*. The infection *Acinetobacter*'s prevalence and the MDR *Acinetobacter* rate were statically higher in the intensive care units compared to other units.
